# Diverse Roles of SIRT1 in Cancer Biology and Lipid Metabolism

**DOI:** 10.3390/ijms16010950

**Published:** 2015-01-05

**Authors:** Glenn E. Simmons, Wendy M. Pruitt, Kevin Pruitt

**Affiliations:** 1 Department of Molecular Genetics, University of Texas Southwestern Medical Center, Dallas, TX 75390, USA; E-Mali: glenn.simmons@utsouthwestern.edu; 2 Department of Immunology and Molecular Microbiology, Texas Tech University Health Sciences Center, 3601 4th Street, STOP 6591, Lubbock, TX 79430-6591, USA; E-Mail: kevin.pruitt@ttuhsc.edu

**Keywords:** sirtuin, SIRT1, metabolism, cancer, lipolysis, lipids, steatosis, fatty acid, sterol regulatory element-binding protein (SREBP)

## Abstract

SIRT1, an NAD^+^-dependent deacetylase, has been described in the literature as a major player in the regulation of cellular stress responses. Its expression has been shown to be altered in cancer cells, and it targets both histone and non-histone proteins for deacetylation and thereby alters metabolic programs in response to diverse physiological stress. Interestingly, many of the metabolic pathways that are influenced by SIRT1 are also altered in tumor development. Not only does SIRT1 have the potential to regulate oncogenic factors, it also orchestrates many aspects of metabolism and lipid regulation and recent reports are beginning to connect these areas. SIRT1 influences pathways that provide an alternative means of deriving energy (such as fatty acid oxidation and gluconeogenesis) when a cell encounters nutritive stress, and can therefore lead to altered lipid metabolism in various pathophysiological contexts. This review helps to show the various connections between SIRT1 and major pathways in cellular metabolism and the consequence of SIRT1 deregulation on carcinogenesis and lipid metabolism.

## 1. Introduction

Mammalian sirtuins deacetylases consist of seven family members (SIRT1-7) that have been shown to be critical regulators of cell signaling pathways. Deacetylases are enzymes that remove acetyl groups from the ε-amino group of lysine residues of histone and non-histone proteins and thereby alter protein function. Deacetylation reactions can take place in the nucleus and cytoplasm, and affect multiple cellular processes [1]. Because some members of the various classes of histone deacetylases (HDACs) have been shown to be overexpressed in diverse cancers, current views suggest that perturbed acetylation patterns on proteins may contribute to cellular transformation and tumor progression [2,3].

The most well studied sirtuin family member, SIRT1, has been shown to alter cellular metabolism and responses to stress and thereby influence programs that direct transcription, apoptosis, autophagy DNA damage repair and senescence [4,5,6]. Influencing diverse physiological processes, it is not surprising that the role of SIRT1 in cellular growth control is complex and its enzymatic activity exerts important cell-type specific effects. This complexity, likewise, extends to studies involving human tumors. For example, SIRT1 expression was found to be significantly associated with shorter overall and relapse-free survival of gastric carcinoma [7], distant metastatic relapse and shorter survival in breast carcinoma [8] and overall survival and event-free survival in soft tissue sarcoma [9]. On the other hand, immunohistochemical analysis of a tissue microarray demonstrated that 23 of 82 carcinomas showed lower SIRT1 expression, and 18 of 82 showed higher expression relative to normal colonic mucosa, indicating the complexity of SIRT1 in tumorigenesis [10]. Additionally, SIRT1 mRNA has been shown to be downregulated in gastric cancer [11].

Other studies have shown that nuclear SIRT1 expression was detected in about 28% of pancreatic ductal adenocarcinoma (PDAC), and expression was found to be significantly higher in poorly differentiated carcinomas. Moreover, strong SIRT1 expression was a significant predictor of poor survival both in univariate and multivariate analyses, further suggesting that imbalances in protein acetylation may influence cancer progression [12].

Overexpression of SIRT1 has been detected in diverse primary solid tumors and hematopoietic malignancies of the breast, colon, prostate, liver and also some types of leukemia [8,13,14,15], while loss of SIRT1 in Sirt1^−/−^ mice is associated with smaller prostates that exhibited a morphologic phenotype similar to that commonly observed within PIN lesions [4]. Due to the fact that SIRT1 activity regulates the function of signaling pathways associated with cell growth and motility [16,17,18], its overexpression could have grave consequences for tumor progression. Conversely, the inhibition of SIRT1/2 was shown to be effective in inhibiting cell proliferation, while inducing apoptosis in cancer cells. These effects have been linked to SIRT1 regulation of several well-established tumorigenic pathways, like Wnt-β catenin and Akt/PI3K [19,20,21]. In this review, we have highlighted reports on SIRT1-mediated regulation of processes involved in lipid metabolism and homeostasis and discuss the implications for tumor biology.

## 2. Lipid Utilization in Tumor Cells

Lipids and sterols play important roles in biological processes in eukaryotes. It is well established that abnormal lipid homeostasis is linked to a number of diseases such as metabolic syndrome, obesity, diabetes, liver steatosis, cardiovascular disease, and cancer [22]. Increased lipogenesis is a well-known hallmark of cancer as lipids, such as fatty acids and cholesterols, can be readily metabolized to provide energy and building materials for the rapidly dividing cells. Cancer cells are said to be capable of producing nearly 95% of their saturated and mono-unsaturated fatty acids *de novo*, even in the presence of adequate dietary supplies [23]. In addition, studies suggest that cancer cells utilize lipolysis, the breakdown of fatty acid, to provide additional raw materials for cellular energetic demands. These processes lead to the systemic dysfunction that is associated with supernormal levels of lipolysis observed in cancer patients [24,25,26].

Producing and breaking down ever-increasing amounts of lipids requires increased lipid mobilization as well. Once fatty acids are generated they must be transported inter-or intra-cellularly between cell membrane and membrane-bound organelles. The fatty-acid-binding proteins (FABPs) belong to a family of proteins that specialize in transporting fatty acids and other lipophilic substances. Thus the FABPs act as lipid chaperone molecules. These family members have diverse functions, but FABP4 in particular, has been shown to be expressed in mature adipocytes, macrophages and dendritic cells which are known for lipid handling [27,28]. However, recent studies have reported that FABP4 is highly expressed in a range of cancer cells and human tumors. For example, one report suggested that adipocytes act as an energy source for the cancer cells, and identified FABP4 up-regulation in omental metastases as compared to primary ovarian tumors. Additionally, FABP4 expression was detected in ovarian cancer cells at the adipocyte-tumor cell interface and FABP4 deficiency substantially impaired metastatic tumor growth in mice. Interestingly, these studies suggest that adipocytes provide fatty acids for rapid tumor growth [29]. Other reports have suggested that the cancer cell itself can create its own internal lipid trafficking. For example, Lee *et al.*, reported that FABP4 expression in the tumor tissue was much higher than that in the non-tumor area of the same specimen and FABP4-knockdown in squamous cell carcinoma cell lines inhibited growth and inhibition of mitogen-activated protein kinase (MAPK) [30]. Further studies from Izumi and colleagues demonstrated that FABP4 treatment promoted serum-induced prostate cancer cell invasion* in vitro* and treatment with FABP4 inhibitor in mice reduced lung metastasis of prostate cancer cells [31]. These reports are examples of the importance of fatty acid binding proteins, such as FABP4, that play an important role in regulating hallmarks of cancer. Thus, not only is the regulation of fatty acid production or lipolysis subject to alteration during tumor progression, but the proteins that handle lipids are also altered. Due to this, factors that are upregulated in cancer cells that can regulate lipid metabolism, such as SIRT1, are attractive targets for study.

## 3. The Role of SIRT1 in Lipid Regulation

SIRT1 regulation of lipid metabolism and its effect on tumorigenesis is an important connection that is becoming increasingly investigated. SIRT1 regulates a cadre of proteins and genes involved in the regulation of lipids. In a study measuring SIRT1 mRNA in adipose tissue biopsies from human volunteers before and after fasting showed an increase in SIRT1 expression in subcutaneous adipose tissue by more than twofold. Fasting or short-term food deprivation has been shown to cause a metabolic shift from lipid synthesis and storage to fat mobilization [32]. These changes are marked by decreases in ATP and NADH, critical cellular metabolites linked to the energetic status of cells. Interestingly, altered levels of NAD^+^, or rather the ratios of NAD^+^/NADH have a profound effect on SIRT1 activity [33]. Also, *in vitro* studies demonstrated that resveratrol, which has been shown to activate SIRT1 in addition to a regulating a number of other targets, significantly enhanced lipolytic effects of epinephrine in human adipose tissue [34]. Also, a role for SIRT1 has been demonstrated in the down-regulation of sterol regulatory element-binding protein (SREBP) orthologs during fasting which results in inhibition of lipid synthesis and fat storage. SREBPs are sterol regulatory element-binding proteins that are transcription factors which remain attached to the nuclear envelope and endoplasmic reticulum membranes until they undergo activation. When cellular sterol levels are low, SREBPs undergo cleavage-induced activation and translocate to the nucleus and promote the transcription of enzymes important for sterol biosynthesis. For example, Walker *et al.*, demonstrated that SIRT1 can directly deacetylate SREBP, and modulation of SIRT1 activity can lead to changes in SREBP ubiquitination, protein stability, and target gene expression. They also showed that chemical activators of SIRT1, such as SRT1720, inhibit SREBP target gene expression* in vitro* and* in vivo*, correlating with decreased hepatic lipid and cholesterol levels and attenuated liver steatosis in diet-induced and genetically obese mice [32]. Interestingly, the SREBP transcription factor family regulates lipid and sterol homeostasis, and SREBPs are highly active in the fed state to promote the expression of lipogenic and cholesterogenic genes and facilitate fat storage. However, under conditions of fasting, SREBP-dependent lipid/cholesterol synthesis is diminished and the contribution of SIRT1 to SREBP-mediated gene expression could potentially become perturbed during tumorigenesis when the tumor microenvironment begins to experience conditions that mimic a fasting-like low nutritive state. Additionally, SIRT1 activation with polyphenols, such as resveratrol, correlated with increased activity of AMP-activated protein kinase (AMPK), another nutrient sensing molecule and corresponding decrease in fatty acid synthesis [35]. Although there are likely many more components to the network of proteins involved in SIRT1-mediated lipid regulation, herein we have focused on a few select targets to demonstrate the breadth of SIRT1 regulation in lipid metabolism.

## 4. SIRT1 as a Regulator of Forkhead Box Protein O1 (FOXO1) and Multidrug Resistance in Cancer

Lipid metabolism is a balancing act of synthesis and breakdown of fat stores for utilization by diverse tissues. Lipolysis is a complicated and multi-step process. Hydrolysis of triglycerides to glycerols and free-fatty acids is accomplished by a series of tri-, di-, and monoacylglyceride lipases [36]. Lipolysis is regulated postranslationally, and the rates of lipolysis are proportional to the cellular levels of adipose triacylglycerol lipase (ATGL), a rate limiting lipolytic enzyme. Both ATGL and hormone-sensitive lipase (HSL) are important enzymes involved in intracellular breakdown of triacylglycerols. ATGL is capable of initiatiating lipolysis and HSL follows and acts on diacylglycerol where both participate in a cooperative fashion for the efficient lipolysis of white adipose tissue. ATGL has been reported to be downstream of SIRT1, and reports show that SIRT1 knockdown decreases basal and isoproterenol-stimulated lipolysis in cultured adipocytes. This effect was attributed in part to the transcriptional suppression of (ATGL) and it was concluded that SIRT1 controls ATGL transcription primarily by deacetylating and activating FOXO1, as ATGL is a FOXO target gene [37].

To appreciate the relationship between SIRT1, ATGL and FOXO1 it is of great utility to understand the importance of FOXO transfactors to cancer biology as a whole. The FOXO family of transcription factors involvement in carcinogenesis is varied depending upon the family member and the tissues involved. For example, FOXO1 has been reported to function as a key regulator of multidrug resistance 1 (*MDR1*) gene transcription. Elevated gene expression of *MDR1* (P-glycoprotein) is a major cause of chemoresistance in many cancer cells and FOXO1 has been shown to be a transcriptional activator of MDR1 in adriamycin-resistant breast cancer cells [38]. Importantly, studies have shown that decreased FOXO acetylation leads to increased nuclear retention of FOXO1 and enhanced expression of FOXO1 target genes [39]. Nuclear FOXO1 is associated with cisplatin and tamoxifen-resistance in gastric and breast cancer cells respectively [40,41]. Additionally, overexpression of SIRT1 with FOXO1 potentiated the transcription of multiresistance protein 2 (MRP2), and the basal activity and expression of SIRT1 was increased in tamoxifen-resistant breast cancer cells. SIRT1 inhibition was reported to reduce both the nuclear FOXO1 levels and MRP2 expression while enhancing cytotoxic effects of paclitaxel and doxorubicin in tamoxifen-resistant breast cancer cells. Interaction of FOXO1 (a direct activator of ATGL) and SIRT1 (an activator of FOXO1) led to activation of FOXO1 which is linked with increased tumorigenicity of cancer cells via acylglycerol kinase [42]. FOXO1, whose activity is increased by deacetylation of SIRT1 [43,44], also regulates thyroid hormone-induced transcription of key hepatic gluconeogenic genes [45].

Interestingly, SIRT1 has been reported to regulate thyroid hormone-induced genes, interact directly with the T3 receptor (TR-β), and contribute to T3-induced regulation of hepatic genes such as CPT1a, PDK4 and SREBP1c [46]. Because of the complexity of SIRT1 loss in animal models and the global involvement of thyroid hormone in regulating metabolism, identifying genes co-regulated by SIRT1 and T3 may prove beneficial. This is important because lipid metabolism is influenced by thyroid hormones such as T3 and a link between SIRT1 and T3-mediated gene expression that influences lipolysis could help reveal cell-type specific contributions of SIRT1. One of the genes regulated by thyroid hormone and SIRT1, carnitine palmitoyltransferase I (CPT1), is a mitochondrial enzyme responsible for the formation of acyl carnitines by catalyzing the transfer of the acyl group of a long-chain fatty acyl-CoA from coenzyme A to l-carnitine. This modification allows for subsequent movement of the acyl carnitine from the cytosol into the intermembrane space of mitochondria. The mitochondrial oxidation of long-chain fatty acids is initiated by the sequential action multiple enzymes including CPT1 and its deficiency results in a decreased rate of fatty acid β-oxidation. Thus, its altered expression resulting from aberrant regulation of SIRT1 could impact fatty acid metabolism. Collectively, these reports establish a connection between SIRT1 regulation of FOXO1, a transcription factor that in turn can influence cell growth and lipid mobilization in cancer cells.

## 5. SIRT1 Regulation of Peroxisome Proliferator-Activated Receptor (PPAR) and PPARγ Coactivator 1α (PGC-1α) Mediated Transcription

Cancer cells require critical transcription programs to up-regulate the numerous pathways needed to sustain pathogenic cell growth. This program is implemented by key transcription factors that can transform cells into metabolically abnormal states that lead to and often sustain tumor growth. One such transcription program is driven by PPAR-γ, which is a major factor in adipogenesis, the mechanism by which preadipocytes differentiate into mature adipocytes. Without PPAR-γ, precursor cells are unable to manifest the characteristic features of adipocytes [47]. PPAR-γ is a critical transcription factor capable of promoting the adipogenic program when over-expressed in mouse fibroblasts, producing fat cells with similar functions to mature adipocytes [48]. Knockout studies further demonstrated the importance of PPAR-γ and linked its involvement with both brown and white fat depots [49] and CCAAT-enhancer-binding protein-α (C/EBP-α), which was also shown to have critical functions in adipogenesis [50]. Gain of function studies revealed that C/EBP-α also initiated adipogenesis, however, unlike PPAR-γ, C/EBP was only required for the formation of white adipose tissue and not brown adipose tissue [50]. Notably, PPAR-γ can initiate adipogenesis in C/EBP-deficient mouse fibroblasts but C/EBP could not initiate adipogenesis without PPAR-γ. Therefore, PPAR-γ is thought to be the dominant player and is also a SIRT1 target. Interestingly, Tian *et al.*, demonstrated that PPAR-γ is deacetylated in a trichostatin-A-senstive and NAD-dependent manner and acetylation-defective PPAR-γ mutants are associated with decreased lipid synthesis in breast cancer cells [51]. PPAR-γ interacts with PGC-1α, which during development regulates brown adipose tissue (BAT) development by acting in conjunction with transcription factors like nuclear respiratory factor 1 (Nrf1). Together Nrf1 and PPAR-γ collaborate to control thermogenesis and mitochondrial biogenesis [52,53]. PGC-1α induces pyruvate dehydrogenase kinase4 (PDK4), which inactivates pyruvate dehydrogenase by phosphorylation and prevents pyruvate entry into the citric acid cycle. PDK4 is localized in the mitochondria and uses its kinase activity to inhibit the pyruvate dehydrogenase complex by slowing the conversion of pyruvate to acetyl-CoA, which allows more conservation of glucose. In addition to being a target gene of the thyroid hormone receptor as discussed earlier, PDK4 is also transcriptionally regulated by other factors such as FOXO1, estrogen-related receptor-α (ERRα), and PPARγ that partner with PGC-1α [54]. Thus, factors that control the fate of acetyl-CoA will also have an impact on lipid metabolism and how malignant cells experiencing limited glucose respond to this nutritive stress.

Interestingly, SIRT1-dependent fat mobilization is mediated through interactions with PPAR-γ cofactors, NCoR and SMRT, which alter the expression of genes associated with adipogenesis including PPAR-γ itself [55]. Also, SIRT1 potentiates the activity of PPAR-α and PGC-1α, leading to increased lipolysis and fat loss in mature adipocytes [55]. This is important because activity of PGC-1α is critical to the activation of the SIRT1-dependent gluconeogenic pathway that is associated with the action of both FOXO1 and hepatocyte nuclear factor 4 α (HNF4-α) [56]. The expression of SIRT1 protein is also associated with protection from hepatic steatosis [57]. In contrast, hepatic-specific knock-down of SIRT1 is concomitant with fatty liver and increased inflammation [58,59]. Both SIRT1 and Wnt signaling have been shown to attenuate adipogenesis, however, only recently was SIRT1/2 shown to regulate Wnt signaling at multiple levels within a cancer context [16,18,19]. For example, SIRT1/2 loss of function was shown to lead to a reduction in Dishevelled (Dvl) protein levels across multiple cancer cell lines [16] In addition to regulating Dvl protein stability, SIRT1/2 was also shown form a complex with Dvl and Tiam1 and promote Rac activation in multiple cancer cell lines [18] While SIRT1/2-mediation regulation of the Dvl/Tiam1 binding was shown to be important for Rac1 activation [18], a separate study demonstrated that SIRT1 also serves as a positive regulator of the Frizzled-7 gene which has been shown to contribute to constitutive Wnt pathway activation [19] Collectively, these studies demonstrate that SIRT1 contributes at multiple levels to the regulation of transcriptional activity of proteins involved in the generation and distribution of fat cells, which is important in influencing lipid homeostasis.

## 6. SIRT1—Mediated Activation of Liver Kinase β-1 (LKB1) and AMPK

**Figure 1:**
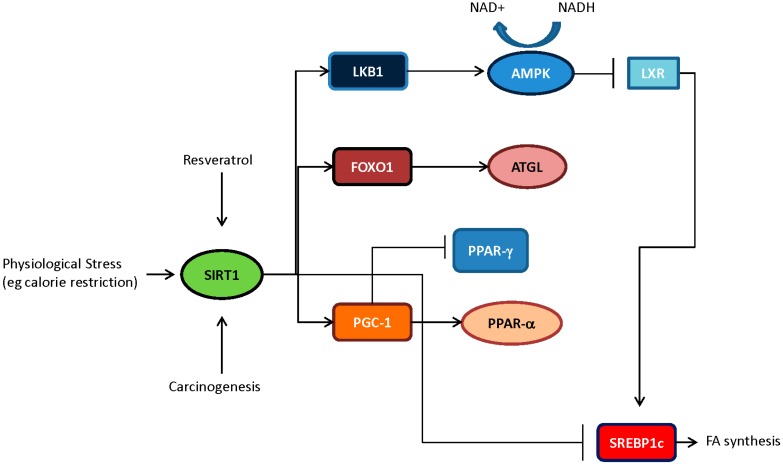
Key lipid regulatory pathways influenced by SIRT1 deacetylase activity. The crosstalk between various signaling pathways allows for SIRT1 activity to function as a potent regulator of lipid homeostasis. Depicted, SIRT1 activation in response to extracellular stimuli, such as caloric restriction or pharmacologic agonist, can lead to the activation of many proteins which have been demonstrated to abrogate lipogenesis by inhibiting master lipid regulator sterol regulatory element-binding protein (SREBP1), while enhancing fat mobilization in cells.

SIRT1 does not only influence nuclear factors, there are also considerable interactions with upstream enzymes that regulate the activity of key cellular pathways. AMP-activated protein kinase (AMPK), for instance is an energy sensor in the cell and is activated upon increases in the AMP/ATP ratio. AMPK is directly activated by phosphorylation carried out by upstream kinases: Calmodulin-dependent kinase kinase β (CamKK), transforming growth factor-activated kinase-1 (TAK1), or by tumor suppressor liver kinase β-1 (LKB1) [35]. However, acetylation of LKB1 at specific lysine residues (K48) regulates kinase activity and the activity of its protein substrates. Deacetylation of LKB1 by SIRT1 potentiates the activity of LKB1 targets such as AMPK. Subsequently, AMPK activity inhibited acetyl-CoA carboxylase (ACC) and fatty acid synthase (FAS) and thus limits the generation of fatty acids [60] (Figure 1). SIRT1 by virtue of its interaction with the LKB1 blocks the synthesis of lipids via the *de novo* synthesis pathway.

## 7. SIRT1 Inhibition of SREBP1c and Fatty Acid (FA) Synthesis

SIRT1 has the ability to further disrupt fatty acid synthesis by perturbing the master regulators of lipid synthesis, SREBP. The sterol regulatory element-binding protein (SREBP) transcription factors (SREBP1a, SREBP1c and SREBP2) are a family of evolutionarily conserved gene regulators that control genes related to the biosynthesis and trafficking of lipids and sterols [61]. SREBP2 is the master regulator of cholesterol biosynthesis and SREBP1c is predominantly involved in regulation of fatty acid (FA) synthesis, while SREBP1a has been implicated in both pathways to some degree [62,63,64]. SREBPs are regulated by proteolytic cleavage that is controlled through a negative feedback mechanism that relies upon the accumulation of lipids and cholesterols produced by the target genes of SREBPs [65,66] and through caloric restriction [67]. The buildup of lipids leads to sequestration of the precursor SREBP molecule in the endoplasmic reticulum preventing translocation of SREBP to the Golgi where SREBP is cut sequentially by two proteases, S1P and S2P, leading to the mature truncated form. The lipid/cholesterol feedback mechanism has been well defined, however the mechanisms by which nutrient deprivation/caloric restriction regulates SREBP1c precursors has remained poorly understood. Mature SREBPs accumulate in the nucleus of mouse hepatocytes following feeding, in contrast to fasting-mediated inhibition of SREBP1 nuclear localization [68]. Giandomenico and colleagues demonstrated that the nuclear/mature form of SREBP1c was acetylated, and that ac-SREBP1 was less ubiquitinated due to competitive binding of acetyl groups to lysine residues. This competitive hindrance results in increased protein stability in the nucleus and thus more expression of lipogenic genes. Conversely, deacetylation of SREBP1 disrupts feeding-induced lipogenesis [69]. SIRT1, which is activated when energy related metabolites (NAD^+^) become limiting in a cell, targets SREBP1 for deacetylation. Correspondingly, pharmacologic activation of SIRT1 decreases the acetylation of SREBP1c, along with the expression of SREBP1c target genes; such as LDLR, HMGR, and FAS, illustrating the antagonistic role of SIRT in the SREBP-mediated lipogenesis pathway [70].

## 8. SIRT1 and Cholesterol Regulation

SIRT1 does not only affect fatty acid generation by SREBP1, but there is evidence that SIRT1 can alter steroidogenesis as well. HMG-CoA synthase (HMGCS) catalyzes the condensation of acetyl-CoA and acetoacetyl-CoA to form 3-hydroxy-3-methylglutaryl-CoA (HMG-CoA) and represents the rate-limiting step in ketone body synthesis. This formation of HMG-CoA represents the first committed, transcriptionally regulated step in cholesterol and isoprenoid biosynthesis. The cholesterol biosynthetic pathway is a proven target for the regulation of serum cholesterol. There are two HMGCS family members that localize to the cytoplasm (HMGCS1) and the mitochondria (HMGCS2), both of which are acetylated on multiple lysine residues. Interestingly, SIRT1 has been shown to deacetylate key lysine residues on HMGCS1, an enzyme that is critical in the synthesis of cholesterol [71]. Because HMGCS1 leads to the production of HMG-CoA, an intermediate in both cholesterol synthesis and ketogenesis, it can be found to be overactive in various pathologies associated with altered lipid homeostasis such as cancer. Therefore it becomes more evident based on the literature that the major pathways involved in the production of lipids in cancer cells are in some way or another regulated by sirtuin activity at a transcriptional or post-translational level.

## 9. Emerging Connections between SIRT1, Estrogen Signaling and Lipid Metabolism

While SIRT1 may impact lipid homeostasis by deacetylating an enzyme early in the isoprenoid and cholesterol biosynthesis cascade, interestingly, it also acts at a very late stage in steroidogenesis. For example, in breast cancer cell lines, SIRT1 has been shown to positively regulate the *CYP19A1* gene, which encodes the aromatase protein. Aromatase is an enzyme that converts androgens to estrogens and is frequently targeted to treat endometriosis [72] and post-menopausal breast cancer [73]. The aromatase enzyme plays a critical role in tumor progression because it increases levels of estrogen within the tumor mass itself and allows tumor growth when the ovaries are producing little postmenopausal estrogen. In fact, aromatase is overexpressed in the majority of breast tumors and endometrial lesions, and aromatase inhibitors (AIs) have been used to successfully mitigate the hyperestrogenic state and block disease progression [74]. Moreover, aromatase over-expression in ERα-negative benign breast epithelial cell lines increases anchorage-independent growth, cell motility, mammosphere formation and induction of depurinating adducts caused by estrogen metabolites, an indication of E_2_–stimulated DNA damage [75,76]. Several studies have demonstrated that aromatase positive tumors show very large increases in intratumoral estrogen levels that may rise to 30-fold the circulating levels in post-menopausal women, suggesting an “intracrine” aspect of this tumor biology [77,78,79]. Interestingly, some of these tumors that were aromatase positive with high estrogen levels were also ERα-negative, suggesting an ERα-independent role of E_2_ [75,79].

Aromatase inhibitors (AI) are frequently used in the treatment of post-menopausal breast cancers because the majority of tumors grow in an estrogen-dependent manner. In support of the importance of targeting hormone-dependent pathways in breast cancer therapy, it is important to note that post-menopausal women with highest quintile of plasma estradiol (E_2_) concentrations present with a significantly higher rate of breast cancer within 10 years compared with the lowest quintile [80,81]. Long-term exposure to estradiol is well documented to increase breast cancer risk and cancer incidence [80,81]. Although how estrogen contributes to malignant progression is still unclear, increasing evidence has shown that higher levels lead to higher genotoxic and mutagenic metabolites of estrogen, expression of stem cell markers and ERα-independent signaling [75,76,82]. In addition, E_2_ contributes to disease progression by both ERα-dependent and ERα-independent mechanisms. The ERα-dependent contribution of E_2_ can stimulate tumor cell proliferation, promote errors during DNA replication, and increase tumor cell survival [75,81,83]. The ERα-independent influence of E_2_ is also known to contribute to tumor progression. For example, E_2_ administration to ERα knockout mice bearing the Wnt-1 oncogene significantly increases mammary tumor incidence, and even in the absence of ERα, aromatase inhibitors decreased tumor formation induced by Wnt-1 [84]. Thus, the hormone-dependence of the majority of breast cancers is pretty clear.

ERα-independent tumors with significant expression may be linked to SIRT1 activity. Several studies have already demonstrated that SIRT1 can influence lipid homeostasis [57,85]. Concurrently, the adiposity of human cells with aromatase deficiency and lipid signatures found in aromatase knockout (ArKO) mice displayed drastically altered lipid profiles as compared to normal controls [86]. Holloway *et al.*, demonstrated that both small molecule inhibitors of SIRT1 and SIRT2, as well as SIRT1-specific inhibition reduce the levels of aromatase mRNA [15]. It was further demonstrated that pharmacologic inhibition of SIRT1/2 caused a marked reduction in aromatase protein levels and SIRT1 immunohistochemistry showed a significant up-regulation in invasive ductal carcinoma relative to normal tissue adjacent to tumor. This work uncovered a novel mechanism for the regulation of aromatase and provides rationale for further investigation of how the inhibition of specific sirtuins may provide a unique strategy for inhibiting aromatase that may complement or synergize with existing therapies. Considering the totality of the previously referenced studies, it is possible that a critical link exists between SIRT1 function, lipid homeostasis and hormone-dependent tumor progression. However, significantly more work needs to be done to firmly establish this connection. In support of this line of reasoning, clinical trials have demonstrated a higher clinical benefit in patients treated with aromatase inhibitors compared to those treated with tamoxifen alone [87] and recommendations have been made to incorporate an AI to reduce the risk of breast cancer recurrence [74]. Therefore, it is also worth further exploring the relationships between sirtuins, altered lipid homeostasis, and aromatase regulation within the context of tumorigenesis.

## 10. Conclusions

Activation of SIRT1 in cells leads to the initiation and suppression of a myriad of processes. SIRT1 as a histone deacetylase is associated with epigenetic mechanisms of gene regulation; however it also has many non-histone/chromatin targets as well. The role of SIRT1 as a regulator of lipid metabolism integrates several metabolic research focus areas, including obesity, diabetes, hepatic steatosis, and cancer. In some cancers, SIRT1 expression is often increased in tumors as compared to benign adjacent tissue. SIRT1 acts upon several transcription factors by activating (FOXO1, PGC-1α) or suppressing (SREBP1c) their activity and producing an overall decrease in the level of lipogenesis in cells. Other factors, including mTOR and autophagy related proteins, are likely involved in lipid homeostasis and further research will be needed to validate the contribution to the SIRT1-dependent mechanisms described herein. However, based on the selected factors described, the overall implications of the research cited suggest that further investigations of SIRT1 may be of interest for clarifying investigate deeper, the roles of lipids in the initiation and progression of human disease. Perhaps, by better understanding the manner in which major metabolic enzymes function in multiple diseases, we will then elucidate the true nature of their function and identify potent targets for therapeutic intervention and disease prevention.
